# Spontaneous rupture of hepatic metastasis as the initial presentation of gastric hepatoid adenocarcinoma: a rare case report and literature review

**DOI:** 10.3389/fonc.2026.1748279

**Published:** 2026-02-02

**Authors:** Bin Zhou, Yingchao Lu, Juan Zhang, Guobiao Yang, Hongxing Xu, Danfeng Shen

**Affiliations:** 1 Department of General Surgery, Suzhou Hospital of Traditional Chinese Medicine Affiliated to Nanjing University of Chinese Medicine, Suzhou, China; 2 Suzhou Medical College of Soochow University, Suzhou, China; 3 Department of Hepatobiliary Surgery, Taicang Affiliated Hospital of Soochow University, Suzhou, China; 4 Department of Physical Medicine and Rehabilitation, Suzhou Hospital of Xiyuan Hospital, China Academy of Chinese Medical Sciences, Suzhou, China

**Keywords:** case report, gastric hepatocellular adenocarcinoma, hepatic metastasis, rupture of hepatic tumor, transcatheter arterial embolization

## Abstract

Hepatoid adenocarcinoma (HAC) is an extremely rare and highly malignant tumor with histological features resembling hepatocellular carcinoma but originating from extrahepatic organs, most commonly in the stomach, known as gastric hepatoid adenocarcinoma (GHA). Spontaneous rupture of hepatic metastasis as the initial presentation of GHA is even rarer, posing significant challenges for clinical diagnosis and management. We present a case of a 74-year-old male admitted to the hospital for right upper abdomen pain. Through a combination of imaging, laboratory tests, interventional therapy, and pathological biopsy, the ultimate diagnosis was confirmed as GHA with spontaneous rupture of hepatic metastasis. The patient was transferred for chemotherapy and immunotherapy following transcatheter arterial embolization (TAE). We systematically reviewed relevant literature and summarized the clinical characteristics, diagnostic methods, treatment strategies, and prognosis of GHA with ruptured hepatic metastases. For patients with ruptured liver tumors, a comprehensive assessment of the potential primary site is essential to avoid misdiagnosis. TAE may create opportunities for subsequent curative surgery, chemotherapy, immunotherapy, and targeted therapy, potentially leading to an overall survival benefit. This case report aims to help clinicians gain a deeper understanding of this rare disease, thereby enabling early diagnosis and optimizing treatment strategies.

## Background

HAC was first described by Ishikura et al. in 1985 (1). It represents a distinct subtype of adenocarcinoma with morphological and immunophenotypic characteristics similar to hepatocellular carcinoma (HCC), but originates from extrahepatic tissues (2, 3). Among HAC cases, the stomach accounts for the vast majority (approximately 70%-80%), followed by organs such as the pancreas, colon, lung, and ovary (2, 4, 5). GHA constitutes only 0.3%-1.0% of all gastric cancers. The lack of specific clinical manifestations makes it difficult to differentiate from common gastric adenocarcinoma or primary hepatocellular carcinoma in the early stages (6). One of the most life-threatening complications of GHA is the rupture of liver metastases, yet similar cases have rarely been documented in the literature. Hepatic metastasis rupture in GHA patients typically results from tumor invasion and destruction of the liver capsule, ultimately leading to intra-abdominal hemorrhage. This often manifests as acute abdominal pain, and if not promptly diagnosed and treated, results in an extremely high mortality rate (7). To date, cases of GHA accompanied by ruptured hepatic metastases are exceedingly rare globally, and no consensus has been reached regarding the optimal diagnosis and management for this condition. This report details a case of a 74-year-old male who experienced a spontaneous rupture of hepatic metastasis due to GHA. Moreover, through a systematic review of relevant literature, we explore the clinical challenges posed by this rare disease and provide guidance for clinical management. This study is compliant with the SCARE criteria (8).

## Case presentation

A 74-year-old male presented to the gastroenterology outpatient clinic with persistent right upper abdominal pain lasting one day. The pain had an insidious onset with no apparent precipitating factors. No radiation to the shoulder or back was noted, and the pain was unrelated to meals. A preliminary diagnosis of peptic ulcer was suspected. The physician prescribed oral rabeprazole 20mg twice daily, which proved ineffective. Later that day, the patient was brought to the emergency department due to a sudden worsening of abdominal pain accompanied by fever, with a maximum temperature of 38.6 °C. Laboratory tests revealed: WBC 4.8×10^9^/L, Hb 92 g/L, hs-CRP 98.6 mg/L, ALT 594.0 U/L, AST 756.0 U/L,GGT 174.0U/L, AFP 2796.96 ng/ml, PIVKA-II 48.96 mAU/mL, with normal levels of CEA and CA19-9. The patient had a past medical history of cerebral infarction and was on secondary prevention medication, taking aspirin (100mg nightly) and rosuvastatin (10mg nightly). Additionally, he had a history of constipation and previous surgeries for intestinal obstruction and appendicitis. There was no history of hepatitis, cirrhosis, or alcohol consumption, nor any family history of hepatocellular carcinoma.

**Figure 1:**
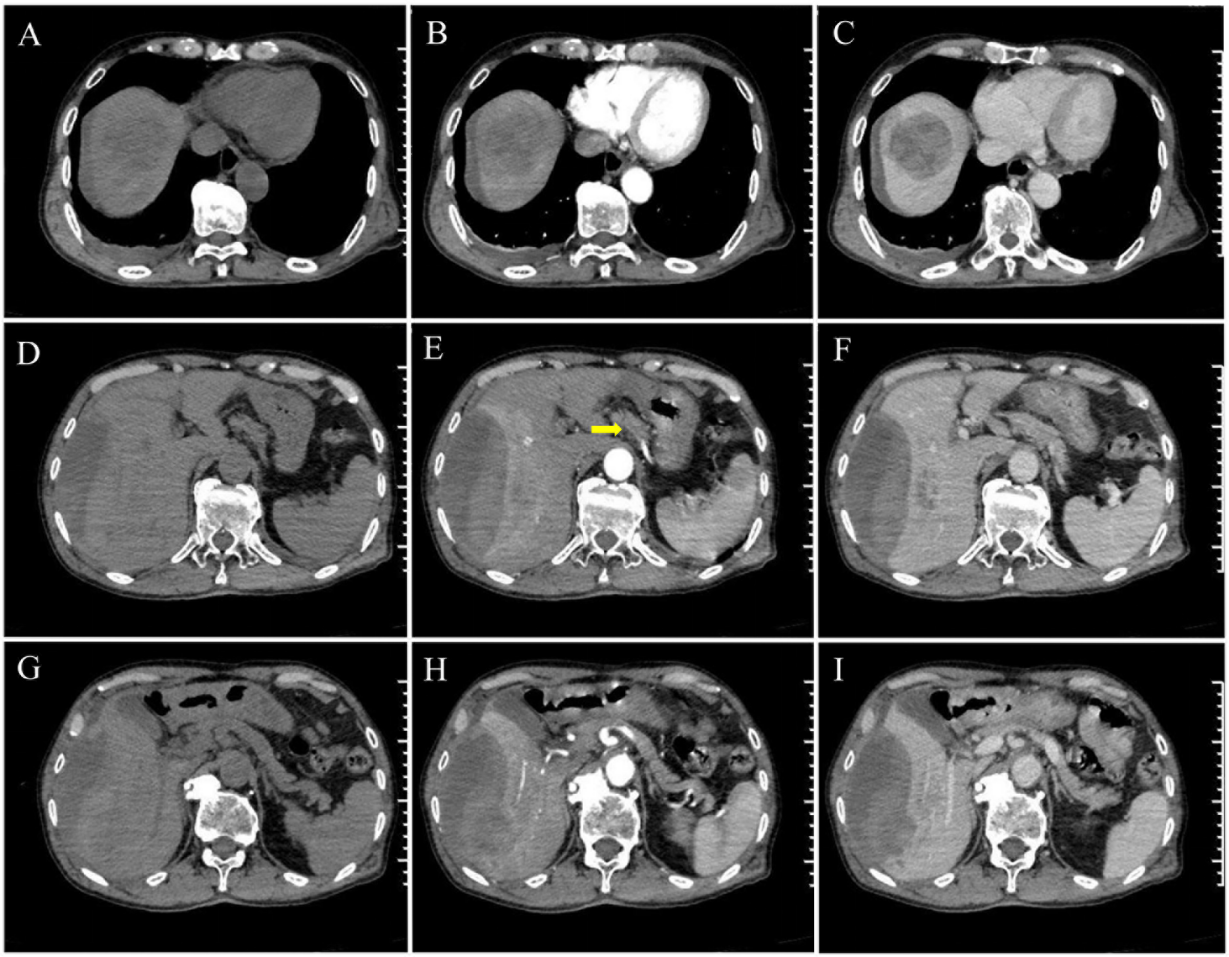
Contrast-enhanced CT images of the GHA patient. **(A-I)** The tumor is located in segments VI-VIII of the liver. **(D-F)** The gastric wall thickening and multiple lymph nodes in the gastric antrum (the yellow arrow). **(D-I)** The ruptured hepatic metastases with hemorrhage and perihepatic effusion.

On the day of admission, the patient underwent an emergency abdominal contrast-enhanced CT scan (GE Revolution CT), which revealed an enormous mixed-density lesion in the hepatic parenchyma and subcapsular regions, indicating the presence of tumor hemorrhage. CT imaging also revealed mild abdominal and pelvic effusion, dilation of the right hepatic duct, and abnormal hepatic perfusion. Additionally, multiple enlarged lymph nodes were visible around the gastric antrum. Preliminary diagnosis suggested a malignant liver tumor, possibly a ruptured HCC (**Figure 1**).

**Figure 2:**
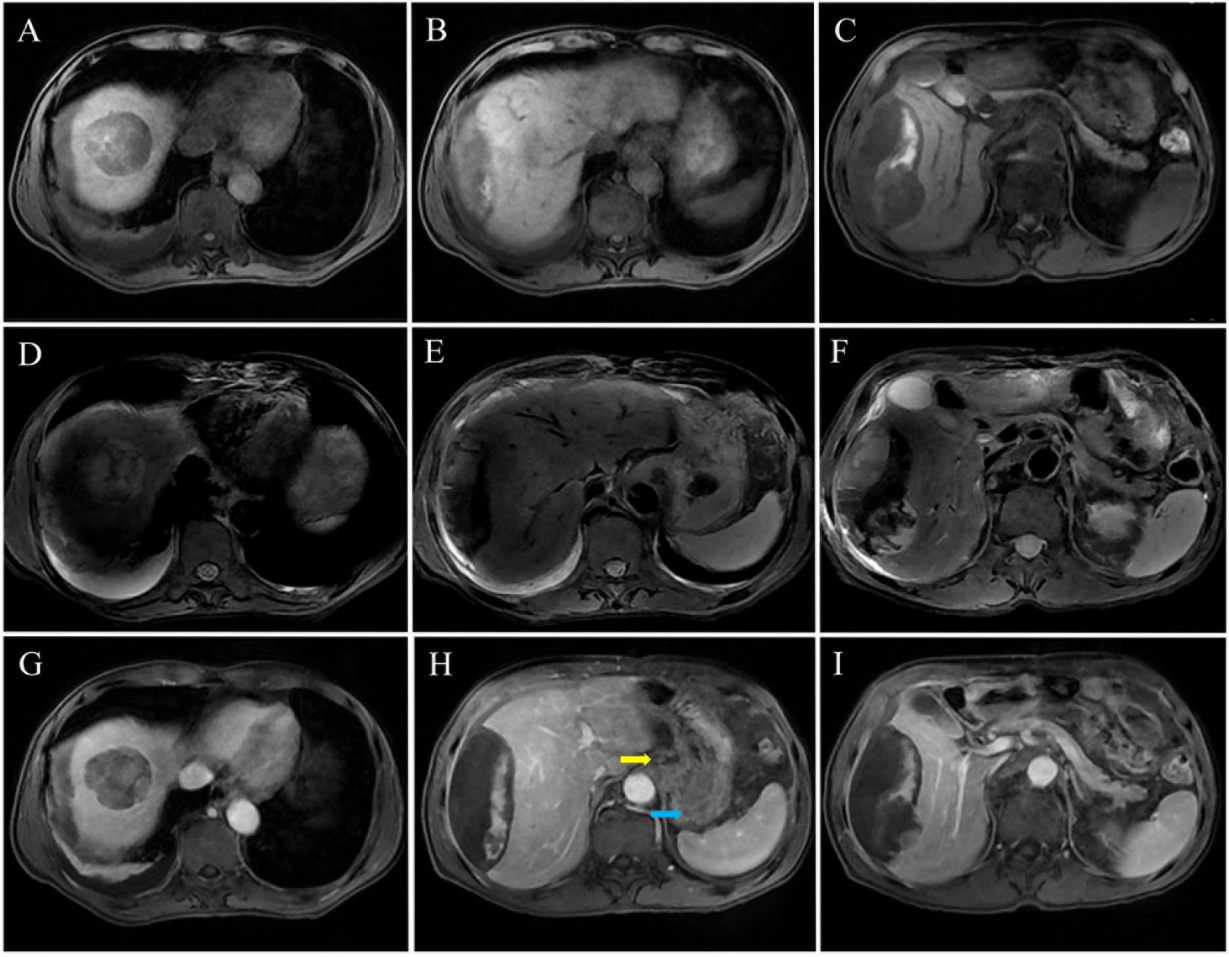
Contrast-enhanced MRI images of the GHA patient. **(A-C)** On T1-weighted imaging, hepatic metastatic adenocarcinoma typically presents as hypointense nodules or masses with signal intensity lower than that of the surrounding normal liver parenchyma. **(D-F)** On T2-weighted imaging, hepatic metastatic adenocarcinoma usually appears as hyperintense nodules or masses with signal intensity higher than that of the surrounding normal liver parenchyma. **(G-I)** MRI findings in the delayed phase showing a typical “bull’s-eye sign”. **(H)** Enlarged lymph nodes adjacent to the gastric antrum (the yellow arrow), with significant thickening of the gastric wall (the blue arrow).

Subsequently, to further confirm the diagnosis, the patient underwent a 3.0T abdominal contrast-enhanced MRI examination, which revealed a malignant tumor in the gastric antrum with local lymph node metastasis and thickening of the gastric wall. Partial abnormal enhancement in the right liver during the arterial phase suggested liver parenchymal compression and edema. The tumor exhibited rim enhancement in the delayed phase with no enhancement in the central necrotic area, considered metastatic lesions with internal hemorrhage and necrosis. Imaging findings combined with markedly elevated AFP levels strongly suggested GHA (**Figure 2**). Given the patient’s hemodynamic stability and absence of shock manifestations, we provisionally adopted a conservative treatment including fluid resuscitation and pain management.

**Figure 3:**
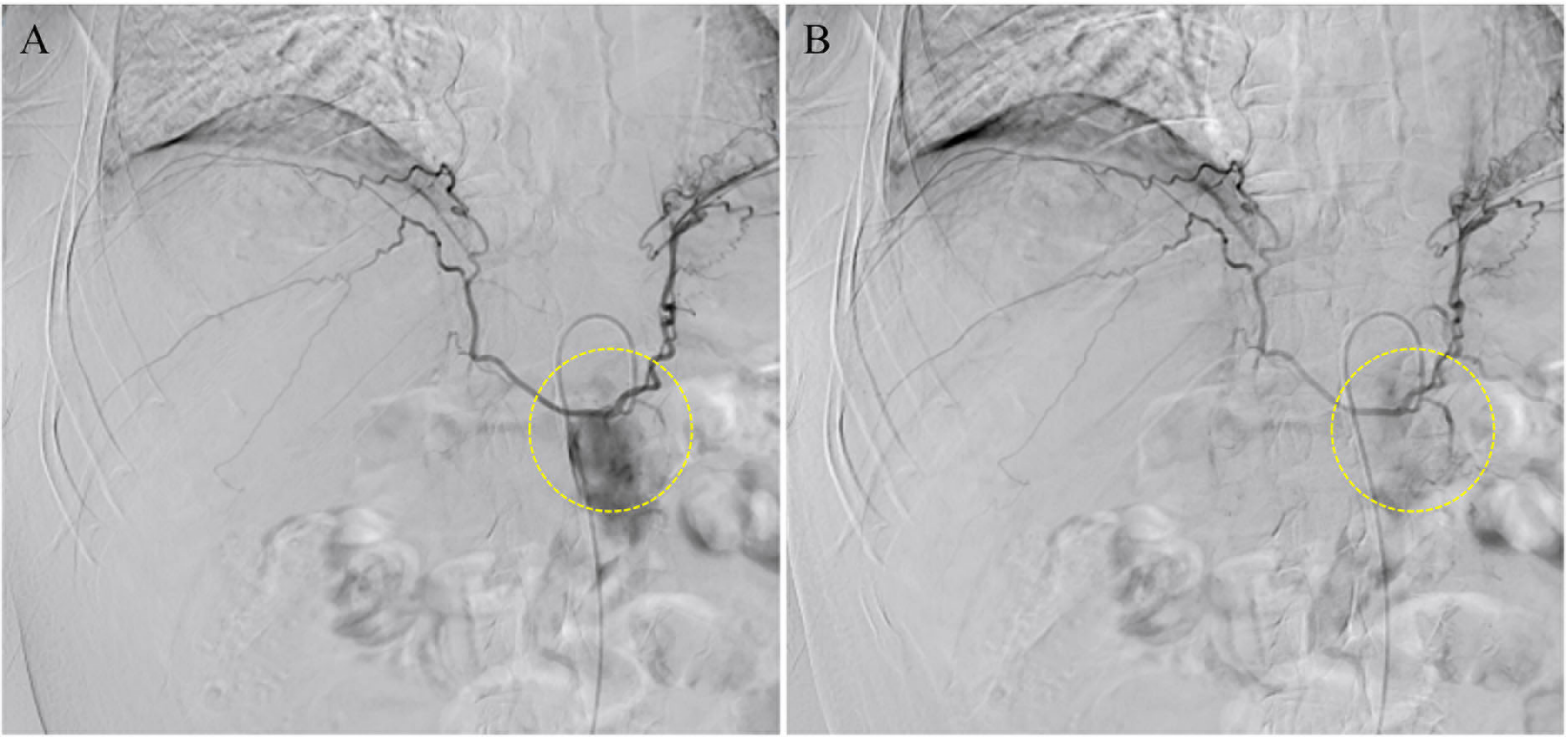
Intraoperative images in TAE obtained via DSA. **(A)** Obvious contrast extravasation (local irregular hyperdense area) is visible, indicating active bleeding. **(B)** The original contrast extravasation completely disappears, and the blood vessels return to normal. TAE has embolized the bleeding artery, achieving the hemostatic purpose. (The yellow dashed circle is the site of arterial bleeding.).

The following day, we confirmed the presence of arterial bleeding within the tumor via digital subtraction angiography (DSA) and conducted nthe TAE therapy. Under local anesthesia with 2% lidocaine, the right femoral artery was punctured using the Seldinger technique, and a 5F vascular sheath was inserted. The coeliac trunk artery was catheterized using a 5F-RH catheter and a hydrophilic guidewire. DSA was performed at a rate of 4 ml/s with a total volume of 20 ml, revealing no abnormal staining in the right liver. Subsequently, a co-axial microcatheter (28MC24130SN, Merrytone) was advanced further into the feeding arteries. Cone-beam CT (CBCT) angiography confirmed that the tumor’s feeding arteries originated from branches of the hepatic artery within segments VI and VIII. The microcatheter was superselected into the tumor-feeding arteries, and a small amount of microspheres (300-500μm, Merrytone) was injected for embolization. Post-embolization angiography showed the disappearance of the tumor-feeding arteries, indicating successful embolization (**Figure 3**). The catheters were then removed, the puncture site was sealed using a vascular closure device, followed by compression with a sandbag and abdominal binder for 30 minutes. Subsequently, the catheter was removed, the puncture site covered with sterile gauze, and continuous pressure applied using a sandbag and an abdominal binder for 30 minutes. After TAE, the patient received anti-infective therapy (Ceftriaxone), fluid infusion (Lactate Ringer’s solution, 0.9% sodium chloride solution, and 5% glucose solution), and liver-protective treatment (Glutathione).

**Figure 4:**
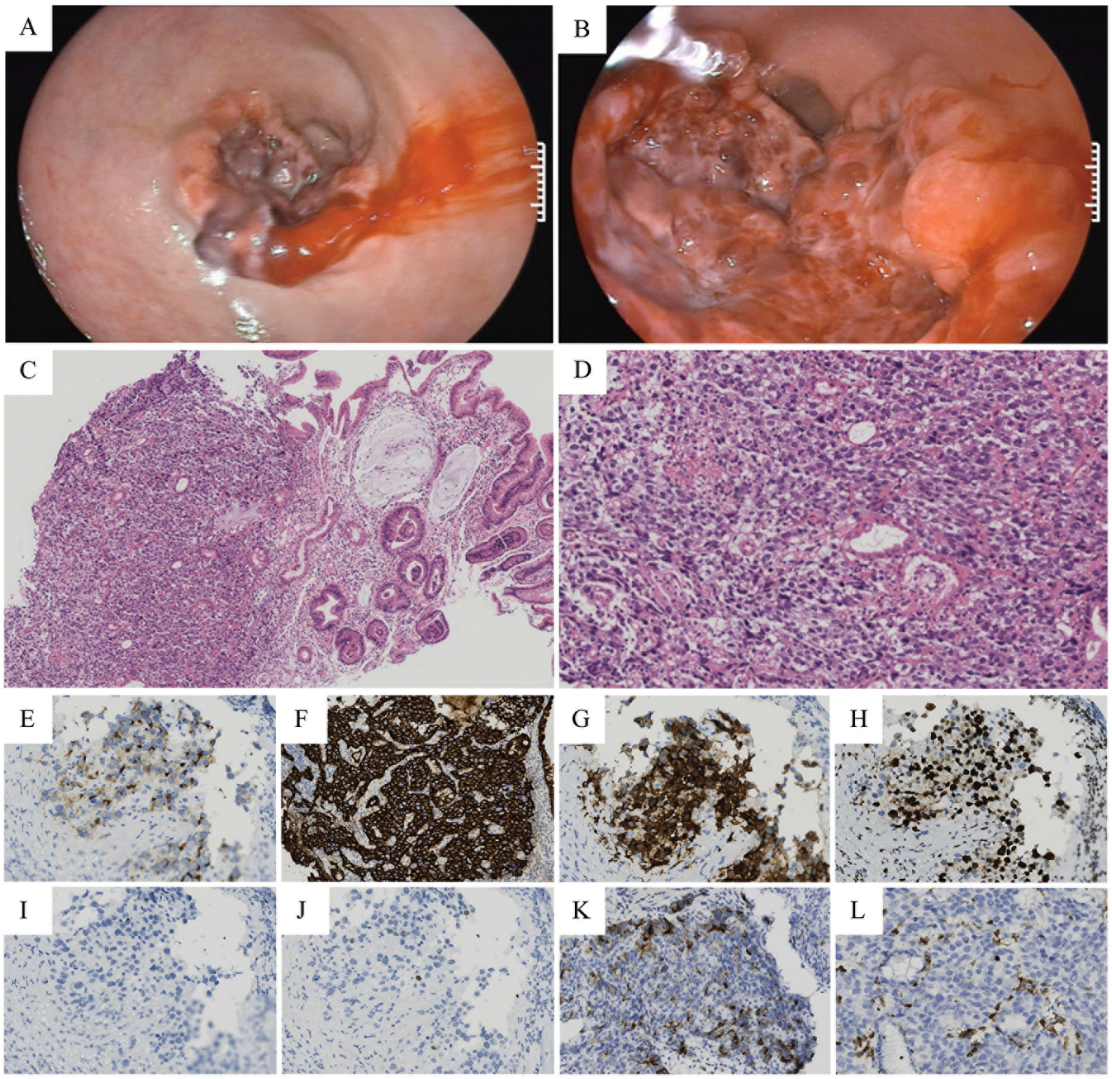
Images of gastroscopy, HE staining, and immunohistochemical staining. **(A, B)** Gastroscopic examination revealed an invasive tumor located in the gastric antrum. **(C)** HE staining (×160). **(D)** HE staining (×400). **(E-L)** Immunohistochemical Staining Characteristics of GHA Cells (×400). **(E)** GPC3 (+). **(F)** CKP (+). **(G)** Villin (+). **(H)** Ki-67 (+). **(I)** HepPar-1 (-). **(J)** CDX-2 (weak +). **(K)** EMA focal (+). **(L)** LCA (-).

On the fourth day of hospitalization, after the patient’s condition stabilized (hemodynamic stability, no significant abdominal pain), a gastroscopy was performed to obtain biopsy samples for pathological examination and genetic testing. Gastroscopy revealed mucosal lesions in the gastric antrum, raising suspicion of malignant tumor in the gastric antrum. Multiple biopsy samples were obtained for pathological examination. Gastroscopic pathological examination and immunohistochemistry revealed: GPC3 (+), CKP (+), CDX-2 (weak +), ki-67 (+), Villin (+), HepPar-1 (-), EMA focal (+), LCA (-). Pathological diagnosis: GHA (**Figure 4**). Next-generation sequencing (NGS)-based genetic testing revealed two Class II variants: CCND1 amplification (copy number: 6) and TP53 exon6 c.578A>G (p.H193R, variant allele frequency: 26.36%). Additionally, the patient’s tumor mutational burden (TMB) was 4.7 mutations/Mb, which was higher than the reference value of 3.6 mutations/Mb. The patient also harbored the TP53 p.H193R variant, and the microsatellite instability (MSI) status was stable (MSS).

After TAE and conservative treatment, the patient’s condition remained stable, with liver function showing improvement compared to admission (ALT 257.0 U/L, AST 139.0 U/L, GGT 91.2 U/L). He was discharged after approximately 2 weeks of hospitalization, and chemotherapy combined with immunotherapy was initiated in the oncology department 3 weeks post-discharge. The treatment regimen included PD-1 anti-tumor immunotherapy plus the mFOLFOX6 regimen (sintilimab 200 mg on day 0, oxaliplatin 0.11 g on day 1, calcium folinate 550 mg on day 1, fluorouracil 550 mg on day 1 plus 3.5 g via continuous infusion for 46 hours, repeated every 3 weeks). To date, the patient has successfully completed the third cycle of treatment, and the patient’s condition is stable accompanied by mild gastrointestinal reactions including nausea and loss of appetite, while the AFP level remains significantly elevated at 1823.19 ng/ml. The patient’s diagnostic, therapeutic and follow-up process is illustrated in **Supplementary Figure 1**.

## Discussion

GHA patients are typically middle-aged to elderly, around 65 years old (male-to-female ratio 2.3:1) (9), which is consistent with the 74-year-old male case in this report. Early symptoms of GHA are often non-specific, such as abdominal pain, loss of appetite, and weight loss, making it easy to confuse with conventional gastric adenocarcinoma (10). However, compared to conventional gastric adenocarcinoma, GHA has a higher tendency for hepatic metastasis and complications such as liver rupture, which may be related to its aggressive biological behavior (11). Diagnosing GHA accompanied by ruptured hepatic metastases is challenging due to its rarity and nonspecific clinical presentation, requiring a comprehensive evaluation based on medical history, laboratory tests, imaging studies, and pathological biopsy. GHA should be differentiated from HCC, as both may present with elevated serum AFP levels. However, GHA exhibits histopathological features of gastric mucosal origin with hepatocellular differentiation, whereas hepatocellular carcinoma (HCC) originates from hepatocytes (6). It also needs to be distinguished from AFP-producing gastric cancer (AFPGC); the latter is defined by serum AFP elevation or immunohistochemical AFP positivity without mandatory hepatocellular morphological features, which is a core diagnostic criterion for GHA (9).

The patient in this case initially presented with right upper quadrant abdominal pain, followed by fever. Physical examination revealed mild tenderness in the right upper quadrant without significant signs of peritoneal irritation. This non-specific clinical picture makes it difficult to distinguish GHA accompanied by ruptured hepatic metastases from other acute abdominal conditions with acute intra-abdominal hemorrhage such as a ruptured HCC. Elevated AFP levels are a typical laboratory feature of GHA (12). This patient’s AFP level was significantly elevated at 2796.96 ng/ml. As a hallmark tumor marker for HCC, abnormally elevated AFP levels in GHA patients often lead to misdiagnosis as HCC, particularly in those with hepatic metastases. In addition to AFP, this patient also had an abnormal elevation of Protein Induced by Vitamin K Absence or Antagonist-II (PIVKA-II). Elevated AFP and PIVKA-II levels are important indicators for diagnosing GHA and other HACs (13). The markedly elevated AFP in this case suggested a possible hepatocytic malignancy, but the absence of a hepatitis history and the MRI finding of a gastric lesion necessitated further investigation for an extrahepatic primary tumor. Furthermore, the patient had anemia (decreased hemoglobin), elevated inflammatory markers (hs-CRP), and abnormal liver function (elevated ALT, AST, GGT), which might be related to tumor infiltration and inflammatory response. Contrast-enhanced CT and MRI are important imaging modalities for diagnosing hepatocellular carcinoma with rupture (3, 14). In this case, contrast-enhanced CT showed mixed-density lesions in the liver with hemorrhage, and MRI further identified a gastric antral lesion and lymph node metastases, providing crucial evidence for diagnosing GHA accompanied by ruptured hepatic metastases. However, it should be noted that the imaging features of GHA complicated with liver metastases are often difficult to distinguish from those of primary liver cancer and metastatic liver cancer. A definitive diagnosis based solely on imaging studies is challenging. Pathological examination remains the gold standard for diagnosing GHA and other HACs. The main pathological features include hepatoid morphological appearance (such as trabecular, nested, or solid growth patterns) and features of adenocarcinoma differentiation (such as gland formation) (15). Immunohistochemical staining is crucial for definitive diagnosis, requiring concurrent positive expression of hepatocyte markers (e.g., AFP, Glypican-3, HepPar-1) and gastrointestinal tumor markers (e.g., CDX-2, Villin) (16, 17). Pathological examination of the gastric antrum biopsy specimen in this case revealed poorly differentiated carcinoma, which was further confirmed as GHA by immunohistochemical staining.

For GHA accompanied by ruptured hepatic metastases, no standard treatment regimen has been established to date (18). Treatment strategies are primarily determined based on the patient’s overall condition, tumor stage, and the extent of hepatic tumor rupture. Ruptured hepatic metastases frequently cause intra-abdominal hemorrhage, a life-threatening complication requiring urgent intervention. Emergency care should focus on stabilizing the patient’s hemodynamics, including fluid resuscitation, blood transfusion, and hemostasis. TAE is an effective minimally invasive method for managing liver rupture caused by malignant tumors, achieving hemostasis and inhibiting tumor growth by embolizing the tumor-feeding arteries (19). In this case, after receiving emergency TAE, the bleeding was successfully controlled, and symptoms were alleviated, laying the foundation for subsequent diagnosis and treatment. Treatment of GHA should be determined based on its location and stage. For resectable GHA with hepatic metastasis, radical gastrectomy with hepatic metastasis resection is the preferred treatment. However, most patients with GHA are diagnosed at an advanced stage with distant metastases, making curative resection impossible. In such cases, palliative treatment options such as chemotherapy, targeted therapy, and immunotherapy can be considered (20–23). The patient in our case was diagnosed with advanced GHA with liver and lymph node metastases, making radical resection impossible. Therefore, we recommended that he undergo chemotherapy and immunotherapy to improve overall survival.

For HAC patients, genetic testing can help identify potential therapeutic targets. For instance, some HAC patients harbor mutations in genes like HER2, EGFR, and MET, and may be sensitive to corresponding targeted drugs (24). Additionally, immunotherapeutic agents such as PD-1/PD-L1 inhibitors have shown efficacy in some HAC cases, though more clinical research is needed to validate their effectiveness (25). Next-generation sequencing (NGS) analysis of the patient in this study identified two Class II variants: CCND1 amplification (copy number: 6) and TP53 exon 6 c.578A>G (p.H193R, variant allele frequency: 26.36%). The patient may be sensitive to drugs such as palbociclib and adavosertib (26, 27); however, the level of evidence is only C/D grade, insufficient to provide conclusive grounds for establishing a therapeutic target. These potential targeted therapies may be indicated as effective (28, 29), but require further investigation in subsequent clinical trials. The patient’s tumor mutational burden (TMB) was 4.7 mutations/Mb (higher than the reference value of 3.6 mutations/Mb), accompanied by the TP53 p.H193R variant and stable microsatellite instability (MSI) status (MSS), suggesting potential benefit from immune checkpoint inhibitor therapy. This is consistent with the research conclusion that high TMB combined with TP53 mutation can predict immune response in MSS patients (30). Nevertheless, the evidence level for targeted therapy drugs is low, and the benefit of immunotherapy is only inferred based on biomarkers. Further evaluation of treatment feasibility requires support from large-sample data in the future.

GHA carries a poor prognosis (31), with a median survival of approximately 7.2 months (32). Early diagnosis and treatment of GHA can significantly improve outcomes. The prognosis for early-stage patients who undergo radical resection is better than for those with advanced GHA. Elevated AFP levels usually indicate a poorer prognosis; patients with higher AFP levels have a greater risk of metastasis and recurrence (33). The liver is a common site of metastasis for GHA. The presence of hepatic metastases significantly worsens prognosis, and the rupture of hepatic metastases further increases patient mortality. Radical resection may be an effective treatment for GHA, with patients undergoing radical resection demonstrating longer survival times compared to those receiving other therapies (34). This patient was diagnosed with advanced GHA accompanied by ruptured hepatic metastases, along with a significantly elevated AFP level. Despite receiving emergency TAE and subsequent chemotherapy and immunotherapy, the patient’s prognosis remains poor, as inferred from the generally unfavorable outcomes associated with GHA.

GHA with hepatic metastasis rupture is extremely rare, and the patient’s long-term use of aspirin may be one of the contributing factors. We conducted a comprehensive search using databases such as PubMed, Embase, and Web of Science. **Supplementary Table 1** summarizes relevant case reports and studies retrieved up to November 2025. Ultimately, 6 cases of GHA with hepatic metastasis rupture were included, summarizing the patients’ clinical characteristics, diagnostic methods, treatment strategies, and survival times. Most cases had significantly elevated AFP levels, and diagnosis primarily relied on pathological biopsy. TAE was the most commonly used emergency treatment for hepatic metastases rupture. Subsequent resection of the primary tumor and hepatic metastases may confer a survival benefit, but the prognosis for patients is generally poor. Literature review Case 2 involved a patient who underwent partial gastrectomy for gastric stump cancer and developed hepatic metastases rupture three weeks postoperatively (34). The patient survived for 6 years (as of the publication date) after hepatic metastases resection, suggesting that the opportunity to perform radical gastrectomy and resection of metastatic lesions may offer potential clinical benefits. Currently, there is no standardized clinical management for GHA accompanied by ruptured hepatic metastases. It is hoped that more cases will be included in the future to provide more experience for the comprehensive management.

## Conclusion

GHA is an extremely rare and highly malignant tumor with non-specific clinical manifestations, leading to diagnostic difficulties. Diagnosis requires the combined use of laboratory tests (such as alpha-fetoprotein, PIVKA-II), imaging studies (contrast-enhanced CT, MRI), pathological biopsy, and immunohistochemical staining. For patients with liver masses of unknown origin combined with elevated AFP, a comprehensive assessment for other potential primary sites is essential to avoid misdiagnosis. When facing emergencies like ruptured hepatic metastases, an individualized treatment plan needs to be rapidly formulated, balancing hemostasis, anti-tumor therapy, and supportive care. More clinical cases need to be accumulated in the future to further clarify its biological characteristics and molecular mechanisms, providing a scientific basis for improving patient prognosis.

## Patient perspective

Three months ago, I experienced ruptured bleeding from liver metastases of hepatoid adenocarcinoma of the stomach. After receiving TAE and conservative treatment, my condition stabilized. Subsequently, I underwent the recommended sequential chemotherapy combined with immunotherapy. Following three cycles of treatment, I had no significant abdominal pain except for gastrointestinal reactions induced by each medication administration. I am fully aware of the rarity and poor prognosis of this disease. During this difficult period, the guidance and care provided by doctors and my family have filled me with profound gratitude. I look forward to the opportunity to proceed to the next phase of surgical treatment, and I would like to express my sincere thanks to the doctors once again.

## Data Availability

The raw data supporting the conclusions of this article will be made available by the authors, without undue reservation.
