# Supplementary material for: Spontaneous rupture of hepatic metastasis as the initial presentation of gastric hepatoid adenocarcinoma: a rare case report and literature review
Source: Front Oncol. 2026 Feb 2;16:1748279. doi: 10.3389/fonc.2026.1748279 (PMC12908172; doi:10.3389/fonc.2026.1748279)
Supplement: Supplementary file 1 [file DataSheet1.docx]

**Spontaneous Rupture of Hepatic Metastasis as the Initial Presentation of Gastric Hepatoid Adenocarcinoma: A Rare Case Report and Literature Review**

**Supporting Information**

**
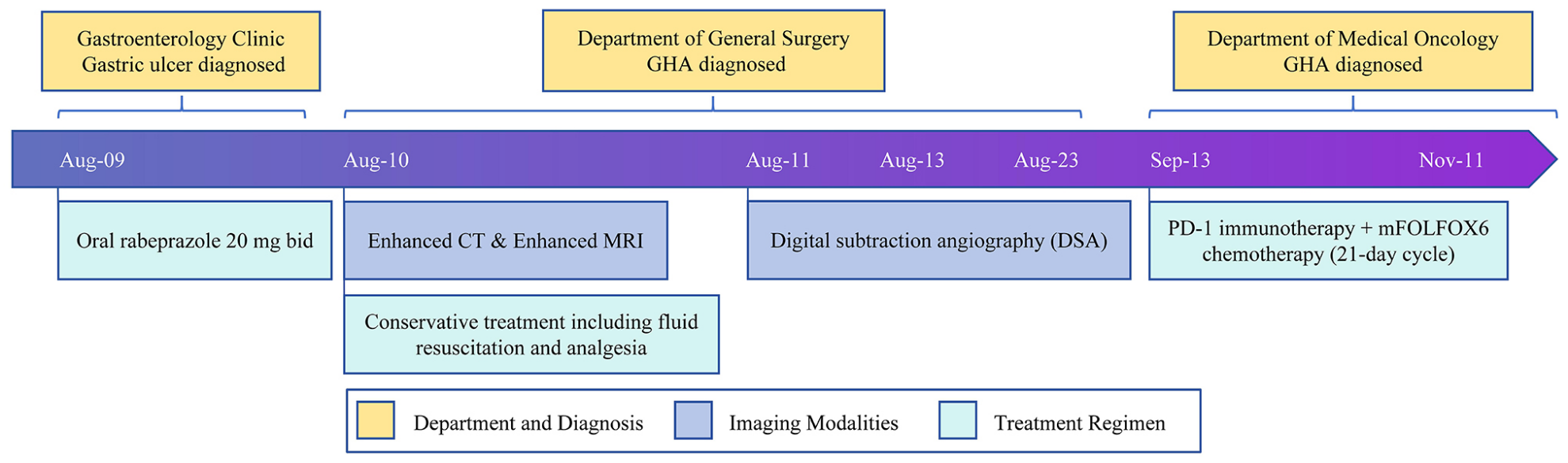
**

**Supplementary Figure 1. The timeline for the patient's diagnosis, treatment and follow-up**

**Supplementary table 1. Summary of clinical data of GHA with hepatic metastasis rupture**

| **Case No.** | **References** | **Age/Gender** | **Primary Site** | **AFP (ng/ml)** | **Diagnostic Method** | **Treatment** | **Survival Time** |
| --- | --- | --- | --- | --- | --- | --- | --- |
| 1 | Yamamoto E et al. [35] | 57/male | Stomach | 4447.9 | Contrast-enhanced CT  Pathology | TAE  Distal gastrectomy  Right hepatectomy  Chemotherapy | 75 days |
| 2 | Yoshida H et al. [34] | 65/female | Stomach | 180000 | Contrast-enhanced CT  Pathology | TAE  Right hepatectomy | 6 years (up to publication) |
| 3 | Kimura M et al. [36] | 60/male | Stomach | 409300 | Contrast-enhanced CT  Gastroscopy  Pathology | Palliative treatment | 27 days |
| 4 | Sohda T et al. [7] | 80/male | Stomach | 21,090 | Contrast-enhanced CT  Pathology | Palliative treatment | Lost to follow-up |
| 5 | Takeyama E et al. [37] | 14/child | Stomach | 556.7 | Contrast-enhanced CT  Pathology | TAE  Right hepatectomy | 4 months |
| 6 | Takeda H et al. [38] | 85/male | Stomach | 411100 | Contrast-enhanced CT  Ultrasonography  Gastroscopy  Pathology | TAE | 39 days |
| 7 | Our case | 74/male | Stomach | 2796.96 | Contrast-enhanced CT  Contrast-enhanced MRI  Gastroscopy  Pathology | TAE  Chemotherapy  Immunotherapy | 98 days (up to the time of manuscript submission) |

**References**

35. Yamamoto, E. *et al.* [a surgical case of AFP-producing gastric cancer discovered by rupture of liver metastatic lesion]. *Gan Kagaku Ryoho, Cancer Chemother.* **45**, 1955–1957 (2018).

36. Kimura, M., Matsuoka, R., Nishikawa, K., Imamura, J. & Kimura, K. Alpha-fetoprotein-producing gastric cancer with ruptured liver metastasis mimicking hepatocellular carcinoma. *Clin. J. Gastroenterol.* **17**, 234–239 (2024).

37. Takeyama, E. *et al.* A case of alpha-fetoprotein-producing gastric cancer in a child presenting with rupture of multiple liver metastases. *Pediatr Surg Int* **31**, 885–888 (2015).

38. Takeda, H. *et al.* [a case of α-fetoprotein-producing hepatoid adenocarcinoma of the stomach with spontaneous rupture of multiple liver metastases]. *Nihon Shokakibyo Gakkai Zasshi = Jpn. J. Gastro-enterol.* **110**, 1625–1632 (2013).
